# Evaluation of treatment response, drug resistance and HIV-1 variability among adolescents on first- and second-line antiretroviral therapy: a study protocol for a prospective observational study in the centre region of Cameroon (EDCTP READY-study)

**DOI:** 10.1186/s12887-019-1599-z

**Published:** 2019-07-05

**Authors:** Joseph Fokam, Maria Mercedes Santoro, Desire Takou, Anne-Esther Njom-Nlend, Paul Koki Ndombo, Nelly Kamgaing, Cedric Kamta, Andre Essiane, Samuel Martin Sosso, Alexis Ndjolo, Vittorio Colizzi, Carlo-Federico Perno

**Affiliations:** 1 Chantal BIYA International Reference Centre for research on HIV/AIDS prevention and management (CIRCB), Yaoundé, Cameroon; 2 Faculty of Medicine and Biomedical Sciences (FMSB), University of Yaoundé I, Yaoundé, Cameroon; 3 National HIV Drug Resistance Working Group (HIVDRWG), Ministry of Public Health, Yaoundé, Cameroon; 4 University of Rome Tor Vergata (UTV), Rome, Italy; 5 National Social Welfare Hospital (NSWH), Yaoundé, Cameroon; 6 Mother-Child Centre of the Chantal BIYA’s foundation (MCC-CBF), Yaoundé, Cameroon; 7 University Health Centre (UHC), Yaoundé, Cameroon; 8 Mfou District Hospital (Mf.DH), Mfou, Cameroon; 9 Mbalmayo District Hospital (Mb.DH), Mbalmayo, Cameroon; 10 University of Milan (UM), Milan, Italy

**Keywords:** HIV, Antiretroviral therapy, Adolescents, Cameroon

## Abstract

**Background:**

Sub-Saharan Africa (SSA) alone has nine out of every 10 children living with HIV globally and monitoring in this setting remains suboptimal, even as these children grow older. With scalability of antiretroviral therapy (ART), several HIV-infected children are growing towards adolescence (over 2.1 million), with the potentials to reach adulthood. However, despite an overall reduction in HIV-related mortality, there are increasing deaths among adolescents living with HIV (ADLHIV), with limited evidence for improved policy-making. Of note, strategies for adolescent transition from pediatrics to adult-healthcare are critical to ensure successful treatment response and longer life expectancy. Interestingly, with uptakes in prevention of mother-to-child transmission, challenges in ART programs, and high viremia among children in SSA, the success rate of paediatric ART might be quickly jeopardised, with possible HIV-1 drug-resistance (HIVDR) emergence, especially after years of paediatric ART exposure. Therefore, monitoring ART response in adolescents and evaluating HIVDR patterns might limit disease progression and guide on subsequent ART options for SSA ADLHIV.

**Objectives:**

Among Cameroonian ADLHIV receiving ART, we shall evaluate the rate of immunovirologic failure, acquired HIVDR-associated mutations, HIV-1 subtype distribution, genetic variability in circulating (plasma) versus archived (cellular) viral strains, and HIVDR early warning indicators (EWIs) at different time-points.

**Methods:**

A prospective and observational study will be conducted among 250 ADLHIV (10–19 years old) receiving ART in the centre region of Cameroon, and followed-up at 6 and 12 months after enrollment. Following consecutive sampling at enrolment, plasma viral load and CD4/CD8 count will be measured, and genotypic resistance testing (GRT) will be performed both in plasma and in buffy coat for participants experiencing virological failure (two consecutive viremia > = 1000 copies/ml). Plasma viral load and CD4/CD8 will be monitored for all participants at 6 and 12 months after enrolment. HIVDR-EWIs will be monitored and survival analysis performed during the 12 months follow-up. Primary outcomes are rates of virological failure, acquired-HIVDR, and mortality.

**Discussion:**

Our findings will provide evidence-based recommendations to ensure successful transition from paediatrics to adult ART regimens and highlight further needs of active ART combinations, for reduced morbidity and mortality in populations of ADLHIV within SSA.

**Electronic supplementary material:**

The online version of this article (10.1186/s12887-019-1599-z) contains supplementary material, which is available to authorized users.

## Background

Sub-Saharan Africa (SSA) alone carries 69.9% of the 36.9 million people infected worldwide and 65.8% of the 1.2 million the global AIDS-related deaths by end 2014, the majority occurring among adolescents [1]. With the highest burden of paediatric HIV infections (2.3 million), about nine out of every 10 children living with HIV found in the SSA region. With about 86.4% of the 220,000 newly infected children worldwide by end 2014 [1], the number of children living with HIV/AIDS will continuously increase overtime, thereby predicting a growing rate of adolescents living with HIV (ADLHIV) in this region. In this line, the burden of HIV/AIDS constitutes a continuous major threat for the future development of SSA countries, and therefore remains a leading poverty-related disease in the entire continent [1–3].

With the advent and scalability of Antiretroviral therapy (ART), there is a global decrease in AIDS-related deaths. Of note, 15.8 million people living with HIV were accessing ART as of June 2015, of which 67.7% were from SSA. Interestingly, about 94% of all children receiving ART are from SSA, a number expected to reach 1.5 million children by 2020 [1, 2]. In this context of continuous new HIV paediatric infections and increasing coverage in paediatric ART, the number of children living with HIV will increase, suggesting a higher likelihood of reaching adolescent age and even adulthood if treatment regimens remain fully effective in controlling HIV infection [2–4]. Most importantly, out of about three million children are living with HIV, an estimated 2.1 million were ADLHIV (10–19 years of age) in resource-limited settings (RLS) by end 2012, with a consistent rising of HIV-related deaths in these ADLHIV even though regressions are seen in other target populations (younger children, pregnant women, adults living with HIV/AIDS) [5]. ADLHIV therefore constitutes an HIV population with growing health concerns and with very limited findings for generalisable best practices specific to this target population, especially in SSA whereby, compared to the western world, there is persistent suboptimal monitoring strategies in routine practice [6–8].

With the increasing scale-up of ART, as high as 20% of children might experience virological failure (VF) in RLS, in the frame of high-levels of viremia generally observed in children. Moreover, as these children grow older, there are increasing risks of treatment failure associated to the development of HIV drug resistance (HIVDR), particularly with the limited therapeutic options available for clinical management [7, 8]. Current paediatric first-line regimens consist of lamivudine, plus abacavir or zidovudine, plus ritonavir-boosted lopinavir (LPV/r) or nevirapine (NVP), depending on the mother-child pair’s history of prevention of mother-to-child transmission (PMTCT) [6]. of note, LPV/r overcomes non-nucleoside reverse transcriptase inhibitor (NNRTI) resistance resulting from PMTCT exposure [2, 3, 9], while NVP matches with current postnatal prophylaxis [3, 8, 9]. The success of this lifelong paediatric treatment will largely depends on the effectiveness of the combination ART from childhood and monitoring overtime [8–14]. Current uptakes in PMTCT interventions and universal ART for children suggest that any infected child stands at high risk of transmitted or acquired HIVDR. Such risks are likely higher in a context whereby: (1) PMTCT practices have been varying overtime (from single dose nevirapine, AZT, to options B and B+), (2) commonly available antiretrovirals are drugs with low genetic-barrier to resistance, (3) drug stock-outs are frequently reported and (4) poor adherence remains challenging. Furthermore, these factors are associated with clinical challenges of which the high viremia in children and the limited laboratory monitoring offer room for delayed detection of treatment failure with accumulation of HIVDR mutations at rates as high as 80% [8, 10, 11, 15–21]. This situation limits therapeutic option as children grow older in SSA, thereby appealing for effective public health strategies for optimal sequencing of ART regimens, in order to maximise long-term treatment success and reduce AIDS-related morbidity and mortality in these RLS [8]. Most importantly, as older children require transitioning from pediatrics to adult ART regimens, sustaining long-term viral suppression prompts the need for reduced risks for mortality among ADLHIV in SSA [5]. With scarcity of evidence that reduces disease progression while preserving next therapeutic options for this growing population of individuals in SSA, there is need to implement an assessment that would guide efficient public health strategies in ART program performance for adolescents [2, 8].

As VF is generally associated with emerging HIVDR, assessing drug resistance mutations (DRMs), in the context of a failing regimen, provides clinical benefit and reduced mortality [9, 10, 22]. An evidence guiding selection of first- and second-line pediatric ART regimens, revealed an urgent need for alternative pediatric treatment options in RLS [23]. Furthermore, Kityo et al. in Uganda revealed high prevalence of pre-treatment HIVDR and a decreasing burden as children grew older (10% of all children versus 15.2% of children < 3 years),implying that adolescents might have archived resistant strains after years of chronic infection [24]. Similar findings from East-Southern Africa reported high-levels of pediatric HIVDR, ranging from 29 to 77% based on PMTCT history, and decreasing rates of DRMs with increasing ages, thus confirming the fading-up of resistant strains over the fitness of wild-type species [11].

As aforementioned, DRMs fade-up as children grow older, and might remain occult in minority viral populations or in the cellular reservoirs [25]. For adolescents vertically infected with HIV, the mechanism of occult HIVDR might be driven by archiving in cellular reservoirs, due to the long-term chronic infection. Of note, our studies among children (aged 1–12 years) with wild type viruses revealed minority DRMs only at early age (1 year), supporting the fact that ADLHIV stand at higher chances of harbouring archived DRMs [26]. Of note, theseoccult resistant variants might remain hidden, ready to repopulate plasma from tissues/reservoirs under pharmacological pressure [11]. The potential presence and the eventual relevance of these minority variants in paediatric settings can be substantial, and merit attention in the frame of limited therapeutic regimens. Demonstrating the clinical implications of such archived DRMs remains an important unmet medical need [26]. In this light, assessing such archived DRMs among adolescents might provide complementary information with an added value for the sequencing of fully active ART combinations, and in turns increase their life expectancy.

When evaluating the efficacy of first- and second-line pediatric ART regimens in RLS, Boerma et al. reported the necessity to set-up salvage pediatric therapies that could be affordable options in RLS [23]. This entails an eventual need of newer second-line or third-line ART regimens. In this line, we earlier demonstrated the absence of major DRMs in the integrase region among ART-naïve, first- and second-line treated patients (including adolescents) in Cameroon, thus indicating a potential efficacy of integrase inhibitors (raltegravir, elvitegravir or dolutegravir) even in heavily treated patients [27, 28]. Furthermore, we also studied CXCR4 and CCR5 viral tropism to determine possible use of anti-CCR5. Our data revealed high rates of X4-tropic viruses among Cameroonian patients, thereby suggesting poor outcomes of CCR5 antagonists (i.e. maraviroc) if used for a public health approach (i.e. without tropism testing) [27–30]. Most importantly, our data from next generation sequencing revealed R5-variants prevailing among children under five years old [26], with increasing proportions of X4-variants, in accordance with previous reports [29, 30]. Thus, pending prospective investigations, a potential salvage ART regimen for adolescents failing ART might contained fully active reverse transcriptase inhibitors (RTIs) and/or protease inhibitors in combination with integrase inhibitor (raltegravir), as an affordable public health approach in RLS [14, 27, 28].

Within the SSA-settings, few studies have addressed the needs of ADLHIV. In South-Africa, a study comparing treatment outcomes between adolescents versus young adults at a public sector community-based ART revealed lower virological suppression rates and higher rates of VF among adolescents [31], thus underscoring the necessity for closer monitoring strategies and the eventual needs of subsequent salvage therapies [23]. In the Central African Republic, an assessment of virologic response and DRMs revealed 34% of children in need of a second-line ART after first-line failure, while 47% of those under second−/third-line ART were experiencing VF with at least one major DRM to RTIs, thus calling for urgent need for improved pediatric ART and monitoring strategies. Among Cameroonian children (aged up to 12 years), we earlier reported high rates (~ 90%) at first-line failure, after a median-time-on-ART of about two years only. Second-line guided by sequencing data showed ~ 70% with virological suppression and ~ 90% with viremia less than 1000 copies/ml. HIV-1 diversity did not interfere with emerging DRMs and CD4 showed a poor sensitivity is predicting treatment response [18]. However, data on medium- to long-term follow-up are completely missing, and recent findings showed potential implications of genetic diversity on virological response, especially among CRF02_AG infected populations [32–35], with considerable programmatic challenges as in Cameroon [36, 37]. While attempting to ascertain AIDS events among adolescents, Sharp et al. reported that, among vertically infected adolescents, the region of HIV-1-Gag targeted by CD8+ T cells as well as the magnitude of CD8+ response may have a clinical importance on monitoring disease progression [38]. With limited evidence in the management of ADLHIV in SSA, it would therefore be of great asset to thoroughly evaluate the immunovirologic response in this target population, in order to identify individual and programmatic factors to be tackled for improved quality of life and life expectancy among ADLHIV. Such analysis will help in tailoring treatment response, resistance profiling and adequacy to subsequent (second- or third-line) ART regimens for ADLHIV in need of newer therapeutic options in SSA.

The READY-Study (*Resistance Evolution among Adolescents in Yaoundé and its surroundings*) aimed at generating information towards improved recommendations for a successful long-term management of ADLHIV in SSA, with adequate and affordable transition policies from paediatric to adult ART regimens in these RLS. Among Cameroonian adolescents (10–19 years old) receiving ART, we shall monitor the therapeutic response to first- and second-line regimens, drug resistance profiling and HIV-1 genetic variability during one-year follow-up. In this population of adolescents receiving ART in Cameroon, we shall: (1) Evaluate the rate of immunovirologic failure in the entire population at enrolment, month-6 and month-12 follow-up points; (2) Evaluate acquired DRMs and HIV-1 subtype distribution among those experiencing VF at enrolment, month-6 and month-12 follow-up points; (3) Evaluate the genetic variability in circulating (plasma) versus archived (cellular) viral strains among those experiencing VF at enrolment, month-6 and month-12 follow-up points; (4) Evaluate HIVDR early warning indicators (EWIs), including survival analysis, in the entire study population through out the one-year monitoring period.

## Methods/design

### Study design

A prospective and observational study will be conducted among ADLHIV receiving ART as per the national ART programme in Cameroon, with follow-up at 6 and 12 months after enrollment.

ADLHIV on ART will be enrolled from four health facilities in the centre region of Cameroon, consisting of:

- Two health facilities from the referral treatment centres (the Mother-Child Centre of the Chantal BIYA foundation and the National Social Welfare hospital, both in Yaoundé);
- Two health facilities from the management units (the Mbalmayo District Hospital in Mbalmayo and the Mfou District hospital in Mfou).

After enrolment (month-0), follow-up will be done at mid-point (month-6) and at end-point (month-12), assessing immunological and virological response, including HIV-1 genotypic resistance testing (GRT) in case of confirmed treatment failure.

### Description of study setting

In Cameroon, a country with a generalised HIV epidemiology (4.3% prevalence in the general population) and a broadly diverse HIV strains (with the prevailing CRF02_AG recombinant) [32–36, 39], the centre region is among sites with high HIV burden (6.1%), and the locality of Yaoundé and its surrounding have a prevalence of 6.3% [40]. HIV prevalence among vertically infected children is still consistent (prevalence of 11.5%) in the country [40], and 1.2% of those aged 15–19 years are infected [39]. Altogether, these suggest a growing rate of ADLHIV in the country, with the centre region being among the most worrying. First-line ART regimens are widely used (96.3%, consisting of two nucleoside RTIs [NRTIs] plus one non-NRTI [NNRTI]) and second-line ART regimens (3.7%, consisting of one protease inhibitor and two NRTIs) are also available nationwide, with a total of 122,638 people receiving ART by end 2013 [41]. The most used regimens for adult are zidovudine plus lamivudine plus nevirapine, followed by tenofovir plus lamivudine plus efavirenz, while for children zidovudine plus lamivudine plus nevirapine and zidovudine plus lamivudine plus efavirenz are predominant [41]. In terms of ART coverage by age range, 4% of treated patients are aged 0–14 years versus 91% aged 15 and above (i.e. 5% unknown age), and adolescents (i.e. aged 10–19 years) are distributed between these two groups [41]. Importantly, the highest coverage of ART is in the centre region (Yaoundé and its surrounding), followed by the Littoral and the North-west regions [41]. The national strategic plan targets 80% ART coverage of children age 0–14 years by end 2015 [42], thus indicating a growing number of adolescents on ART.

The choice of the Centre region for the present study in Cameroon is therefore based on its high HIV burden, its high coverage of ART, the long-term experience in ART provision of health facilities [42], as well as the wide HIV-1 genetic diversity reported from vertically infected children and adolescents living in this geographical setting [18].

### Sampling method

A consecutive and non-randomised sampling method will be used for participant enrolment, according to eligibility criteria.

#### Eligibility criteria

##### Inclusion criteria

Will be enrolled in the study ADLHIV (aged 10–19 years), who are receiving a first- or second-line ART for ≥6 months in one of our study sites, who provide their written assent, whose legal guardian accept to provide a written informed consent (in addition to written assent provided by the adolescent), and irrespective of PMTCT antiretroviral history (exposed to PMTCT drugs, not-exposed to PMTCT drugs, or unknown exposure).

##### Non-inclusion criteria

Will not be considered for enrolment ADLHIV not found (i.e. not formally registered) in the ART monitoring system of a study site, ADLHIV reported to be ART-naïve, to be on a drug regimen not included in the national guidelines, or to be on a structured treatment interruption.

##### Exclusion criteria

Will be considered excluded from the study any participant who decides deliberately/freely to withdraw in the course of the study, or transferred out of a study site before mid- or end-point.

#### Sample size

Assuming the rate of VF at 40%, a 95% confidence and 80% statistical power, sample size is 174 patients. Adding 10% potential LTFU during the one-year study period and 20% sequencing failure rate [10, 18], minimal sample size is 243 ADLHIV, rounded-up to 250 total sample size, further stratified into 150 ADLHIV in the referral centres (75 per site) and 100 in the HIV management units settings (50 per site), as per coverage in ART in these two geographical locations [41, 42].

#### Sampling strategy

All ADLHIV meeting study criteria will be invited for a written assent, as well as their legal guardians for a written informed consent. Fully consenting participants will be consecutively enrolled until complete sample size per site (75 for each of the two HIV referral centres and 50 for each of the two HIV management units). All participating ADLHIV will be consecutively assigned a unique ID number per site, and data will be centralised at the International Reference Centre Chantal Biya (CIRCB) for research on HIV/AIDS prevention and management in Yaounde, Cameroon. Physical and telephone contact details of participants will also be registered in the database for active follow-up, whenever necessary, by the study community-relay agents.

### Study procedures and variables

#### Procedures and timeline

Entire schedule requires 30-months overall: 6-months (month1–6) for administration/ethics/field preparedness, 6-months (Month 7–12) for enrolment of participants until complete sampling, 12-months monitoring (Month 7–24) for complete follow-up of one-year, and 6-months (Month 25–30) for closing study sites, data processing, and reporting. During enrolment (M0) at the clinic, study clinicians will obtain informed consent and participant assent from legal guardian and the ADLHIV, respectively, followed by collection of socio-demographic, clinical and economic data, HIV history (including date of HIV diagnosis and WHO-clinical staging), complete history of ARV (ART and/or PMTCT, previous viral load and CD4/CD8 whenever available), as well as adherence levels. Intravenous blood (6 ml) will be collected from each ADLHIV for laboratory analyses at the CIRCB. Practically, CD4/CD8 (cells/mm [3] and percentage) and plasma viral load (PVL) will be performed for all ADLHIV using flow cytometry and real-time polymerase chain reaction (PCR) respectively. Among those experiencing VF, RNA (from plasma) and DNA (from buffy coat) extracts, isolated using the QIAamp commercially available Viral RNA and DNA Mini Kit (QIAGEN), be will be used for PCR-amplification of the protease-reverse transcriptase (PR-RT) region of HIV-1. GRT will be performed on PR-RT amplicons using Sanger-sequencing, as previously described [43]. HIVDR-associated mutations will be interpreted using *Stanford HIVdb* algorithm [18, 35, 36, 43]. GRT results will then guide the selection of effective ARVs available in the national ART programme (first-, second-, and third-line regimens) and outcomes (WHO-clinical staging, viral and CD4/CD8 measurements) will be measured at 6 and 12 month-visits.

Follow-up visits will be at mid-point (months Month 6 [±1]) and at endpoint (Month 12 [±2]) after enrolment into the study. At each time-point (baseline, mid-point and end-point), epidemiological and clinical data will be collected using the same procedure described above during enrolment (i.e. baseline), including ART-adherence assessment. 6 ml of intravenous blood will be collected per visit (for CD4/CD8 and PVL). Among those experiencing VF at these visits, GRT will be performed as described above [18, 35, 36, 43], and the results will then guide the selection of effective ARVs available in the national ART programme for first-, second-, and third-line regimens.

At each study phase (enrolment, midpoint, endpoint), residual samples will be stored at − 80° available in the blood bank of the CIRCB. The entire work plan requires 30-months: 6-months (month1–6) for administration/ethics/field preparedness, 6-months (Month 7–12) for enrolment of participants until complete sampling, 12-months monitoring (Month 7–24) for complete follow-up of one-year, and 6-months (Month 25–30) for closing study sites, data processing, and reporting.

### Detailed procedures

At month-0, enrolment will be done at study clinic for all adolescents meeting study eligibility criteria. Participants will then be followed up during 12 months after enrolment, with scheduled visits at the clinic at months 6 (±1 month) and 12 (±2 month), for a total of 3 scheduled visits. Patients with virological failure (viral load > 1000 copies/ml) at any timepoint will undergo an extra visit in the next 1 month with adherence support and to confirm the virological failure (VF) as aforedescribed. Participants’ clinical management not considered in the study protocol will follow local recommendations for clinical practice at the discretion of the responsible physician.Procedures at Baseline visit (month 0). Mothers will be required to provide data contained in the Baseline Case Report Form (see clinical data definition below). Briefly, this will cover detailed epidemiological and clinical information, including socio-demographic and economic data, HIV history, history of antiretroviral usage by the adolescent, including drugs taken as part of PMTCT (if applicable), adherence to ART, and results from any laboratory test that were performed before study enrolment and available in the patient medical record file. Blood will be collected from each participating adolsecent, for a total of volumen 6 ml per timepoint for the following: determination of: viral load (600 μl plasma); CD4/CD8 levels (100 μl whole blood); HIV-1 Sanger sequencing for protease-reverse transcriptase (1000 μl plasma for RNA extraction and 200 μl buffy coat for DNA extraction); residual plasma samples will be stored at the biobank.

Laboratory procedures on sample management plans are provided below. Procedures at follow-up visits at months 6 and 12. Participants attending follow-up and their legal gauardians will be required to provide data contained in the follow-up Case Report Form, for updated information on epidemiological and clinical situation, with special emphasis on antiretroviral treatment information and HIV-related clinical events. Blood samples will be collected as described above. Similarly, for plasma viral load > 1000 copies/ml, adherence support will be provide then GRT performed if and only if virological failure is confirmed one-month later.All laboratory analysis will all be performed at the International Reference Centre Chantal Biya (CIRCB) in Yaounde, Cameroon, among which: Viral load measurementsWill be performed using Abbott m2000rt, as per the manufacturer’s instructions. Briefly, 600 μl of plasma collected from EDTA tube is used for viral RNA extraction. Based on the principle of reverse transcription polymerization (RT-PCR), amplicons are generated from the RNA genome of HIV-1 in clinical specimens. An RNA sequence that is unrelated to the HIV-1 target sequence is introduced into each sample at the beginning of sample PCR mix preparation. This unrelated RNA sequence is simultaneously amplified by RT-PCR, and serves as an internal control (IC) to demonstrate that the process has proceeded correctly for each sample. The amount of HIV-1 target sequence that is present at each amplification cycle is measured using fluorescent-labeled oligonucleotide probes on the Abbott m2000rt instrument. The amplification cycle at which an increase in fluorescent signal is detected by the Abbott m2000rt is proportional to the log of the HIV-1 RNA concentration present in the original patient sample.CD4/CD8 will be performed using FACSCalibur, as per the manufacturer’s instructions. Briefly, determination of lymphocyte subsets (CD3, CD4, CD8) will be performed on the flow cytometer (BD Biosciences, San Jose, CA) using a single-platform technology. A volume of 50 μl whole blood will be stained with 10 μl of Multitest reagent in Trucount tubes for 15 min (all from BD Biosciences), and red blood cells will be lysed by using fluorescence-activated cell sorting lysing solution (BD Biosciences). Data acquisition will be performed using CellQuest-Pro software (BD Biosciences) creating up to nine multicolor acquisition dot plots that use eight multicolor gates. Data will be then analyzed with FlowJo software version 7.5.5 for Microsoft (TreeStar, San Carlos, CA). Using Multitest reagents with Trucount tubes in the laboratory procedure, absolute subset count will be calculated with the following formula: (number of events in the region containing the cell population/number of events in the absolute count bead region) × (the number of beads per tube/test volume). If ncessary, a FACSCanto II (alternative platform to FACSCalibur) could be used for the same CD4/CD8 measurement purposes. Briefly, these analyses will be performed using the FACS Diva software (BD Biosciences, San Jose, CA), by reading information from the BD FACSTM 7-color setup beads label into BD FACSCanto clinical software (2D barcode symbology), and also by reading coded patient information into a worklist. Sanger Sequencing for HIV-1 drug resistance testing will be performed for adolescent experiencing virological faiure, using ABI 3130xl (Genetic Anlyser with 16 capillaries), HIV genotyping will be performed using plasma samples and an in-house reverse transcriptase polymerase chain reaction (PCR) protocol, as previously described (Fokam et al., 2011). Briefly, RNA will be extracted from 140 μl of concentrated plasma using the QIAamp Viral RNA minikit (Qiagen, Milan, Italy), according to the manufacturer’s protocol. RNA extracts will then be retrotranscribed and amplified using the kit One-Step Invitrogen (Foster City, CA) (SuperScript One-Step for long templates RT-PCR) and 2 different sequence-specific primers for 40 cycles. For insufficiently amplified samples after the first round PCR, a second round PCR (semi-nested PCR) will be performed. A direct sequencing reaction will be done using 7–8 overlapping primers. For a full plate run, the sequencer of 16 capillaries will analyse 2 different patient samples simultaneously for ~ 2.5 h per run, and assembly program (Seqscape v2.7) will be used for sequence analysis, and mutations will be interpreted following the Stanford HIVdb algorithm. Therefore, 12 samples will run for 15 h in total. Study timeline is provided as Additional file 1: (SDC) 1.

**Table:** 

EWI and title	Definition	Numerator	Denominator	Target
EWI_1_ On-time pill pick-up	Proportion of adolescents that pick-up ART no more than two days late at the first pick-up after the baseline pick-up.	Number of adolescents picking-up their ART “on time” at the first drug pick-up after baseline pick-up date	Number of adolescents who picked-up ARV drugs on or after the designated EWI sample start date	Desirable performance (green): > 90%Fair performance (amber): 80–90%Poor performance (red): < 80%
EWI_2_ Retention in care	Percentage of adolescents known to be alive and on ART 12 months after enrolment	Number of adolescents who are still alive and on ART 12 months after enrolment	Total number of adolescents (excluding transfers out) who were expected to achieve 12-month outcomes	Desirable performance (green): > 85%Fair performance (amber): 75–85%Poor performance (red): < 75%
EWI_3_ Pharmacy stock-outs	Percentage of months in the study period in which there were no ARV drug stock-outs	Number of months in the designated year in which there were no stock-out, days of any ARV drug routinely used at the site	12 months of the reporting period	Desirable performance (green): 100%Poor performance (red): < 100%;
EWI_4_ Pharmacy dispensing practice	Percentage of adolescents being dispensed a mono- or dual-ART	Number of adolescents who pick up from the pharmacy, a regimen consisting of one or two ARVs;	Number of adolescents picking up ART on or after the designated EWI sample start date	Desirable performance (green) defined as 0% adolescents picking-up a mono- or dual-ARTPoor performance (red) defined as > 0% adolescents picking-up a mono- or dual-ART, and
EWI_5_ Virological suppression	Percentage of adolescents receiving ART of ART whose viral load is < 1000 copies/ml 12 months after enrolment	Number of adolescents receiving ART 12 months after study enrolment and whose viral load is < 1000 copies/ml	Number of adolescents who have a viral load performed at 12 months after enrolment	Desirable performance (green): > 85% Fair performance (amber): 70–85%Poor performance (red): < 70%

### Abstraction of HIVDR EWIs

Data on HIVDR EWIs will also be collected at each study phase, consisting of five variables among which EWI_1_ (*on-time pill pick-up*), EWI_2_ (*retention in care*), EWI_3_ (*pharmacy stock-outs*), EWI_4_ (*dispensing practices*), EWI_5_ (*virological suppression*). Among patients not retained in care during the 12-month follow-up period, we shall investigate to the underpinning reason that might be deliberate ART interruption, transfer out or mortality. Cases of mortality will then serve to determine the survival rate among the study participants.

#### Definition of study variables

- VF will be defined as two consecutive PVL ≥ 1000 copies/ml within 1 month under intensive adherence counselling and support [10]. Participants with PVL ≥ 1000 copies/ml at any visit will therefore undergo adherence counselling and extra-visit after 1 month to confirm or infirm VF.
- Immunological failure will be defined as persistent CD4 < 100 cells/mm [3] during follow-up.
- Clinical failure will be defined as WHO clinical stage 3 or 4 events or death. Clinical management outside the study protocol will be done according to local national guidelines.
- HIVDR will be defined as the presence of any mutation, in the PR-RT regions of HIV-1, known to reduce the efficacy of NRTI, NNRTI or protease inhibitor, as per the *Stanford HIVdb* algorithm [18, 35, 36, 43].
- Good adherence will be defined as ≥95% compliance to ART (i.e. only one missing dose), measured by pill count and by self-reported, during the past 30 days.
- EWIs will be defined and their respective performance targets are provided below: [10, 3, 8]

### Quality assurance

For each of the 3 study phases (enrolment, midpoint, endpoint), quality assurance will be performed by proficiency testing for PVL, CD4/CD8 (cell/mm [3] and percentage) and Sanger-based GRT.

### Management of potential study risks

Lost to follow-up (LTFU) is a potential risk in reducing the power of this study for evidence-based recommendations. To limit LTFU, adherence support will be intensified and the site community relay agents will undertake active search of defaulters.

Investigators’ compliance may be concerning at certain points of the study (i.e. if a collaborator moves for several reasons). To handle this potential challenge, site visit will be conducted weekly and site compliance to study regulations will be assessed by monthly supervision. The lead applicant will regularly coordinate activities with study-site clinicians and with guidance from mentor.

Data protection and safety might be concerning. This will be handle by procurement of a new computer device and a specific database dedicated exclusively for the project, with encrypted password and a protected working station. Potential bias in laboratory data will be handled with quality assurance. To ensure functionality of equipment, routine preventive maintenance will be ensured. If for any unforeseen reason an equipment breakdown, the backup laboratory (Centre Pasteur du Cameroun) will covered the required period, under current agreement. For staff protection, biosafety will be ensured by use of personal protective equipment as per international standards, including use of for a biosafety level II cabinet for project related-laboratory activities.

### Data collection and analysis

Data will be collected per site and stored in a password-protected database at the CIRCB, Yaoundé-Cameroon, whereby the project statistician will coordinate data management and analyses using SPSS software. Study data will consist of sociodemographic, clinical and laboratory information. Data Management will be under the responsibility of the project Biostatistician of the CIRCB in Younde-Cameroon, in coordination with the principal investigator. Data management will therefore entail procedures for sociodemographic, clinical and laboratory data collection, data protection, data quality assurance and data analysis. This procedure will be similar at enrolment (M0), midpoint (M6) and endpoint (M12). Standardized Case Report Forms (CRFs) will be generated and provided to the four study sites for on-site collection of data. In addition to basic demographic information, CRFs will include all clinical information, as well as information on type of sample collected, including date, number of aliquots, volume of aliquots and sample identification numbers to reassure further tracing of laboratory results. CRFs will be translated and provided in French and in English as per national regulations in Cameroon. However, mainly the French version will be used as the study sites are all within the French-speaking sector of the country. CRFs will be completed by the project site collaborator, and double-checked by a research fellow for consistency, if necessary, the CRF will be correctly completed and signed by the site principal investigator or other authorised person. At the end of each study visit, double-checked and signed CRFs will be sent to data entry. Laboratory CRFs will be also generated and provided in paper to the corresponding local laboratories to introduce laboratory results, together with identification number of samples. At the completion of laboratory procedures, laboratory project staff will be responsible for completion of laboratory CRFs, which will be double-checked by the co-investigator for laboratory analyses and, if necessary, the CRF will be correctly completed and signed by the site principal investigator or other authorised person. At the end of this process, double-checked and signed laboratory CRFs will be sent to data entry.Data collected through clinical and laboratory CRFs will be entered on site through double entry, by designated data entry personnel. A specific database will be created for the protected entry of clinical data and laboratory results. Data storage will be done in numerical codes generated consecutively per site. Samples for plasma viral load, CD4/CD8, and eventually for genotypic resistance testing (in case of treatment failure) to be daily centralised for analysis at the CIRCB laboratory, using participant anonymous ID code for their respective sample, alongside the corresponding sample transportation form. The laboratory co-investigator at CIRCB or other authorized person will have full responsibility for the completion of any procedures.

At enrolment, midpoint and endpoint, prevalence of VF and HIVDR (with 95% Confidence Intervals) will be described globally, by geographical settings and by site. Associated factors will be evaluated using multivariate logistic regression, with an estimative approach for the unbiased effect of ART regimens (first versus second-line) and HIV-1 diversity (CRF02_AG versus non-CRF02-AG) [18]. In order to provide the highest level of evidence amongst ADLHIV experiencing VF versus those on virological success, ART response will be described through time to event data analysis and mixed models for the trajectory of CD4 count and viral load, based on adjustments of baseline confounders (duration of treatment, age, gender, orphan hood, site). The effect of DRMs (from plasma versus buffy coat) on ART response will be evaluated using multivariate survival models, either Cox or Fine&Gray in the presence of death as competing event. Characteristics of patients LTFU will be described and, if necessary, inverse probability weights will be used. Primary outcome is the proportion of VF; secondary outcome is the proportion of acquired-HIVDR, or deaths, evaluated according to study site (referral centres versus management units), HIV-1 subtype distribution (CRF02_AG versus non-CRF02-AG) [18], adherence levels (< 95% vs. ≥95%) [44], and ART regimens (first versus second-line) [35]. Proportion of adolescents with low-level viremia (≥400 or ≥ 50copies/ml) who finally end in VF will be described, along with HIVDR profile at endpoint [45]. We will build a multivariate logistic model with predictive approach to evaluate the association of socioeconomic conditions with adherence levels and HIVDR emergence [44, 46].

## Discussion

Our research objectives will help in generating evidence for optimal monitoring and management of adolescents treated with antiretroviral therapy (ART) in a sub-Saharan African (SSA) context. Specifically, study objective 1 will help in understanding the significance of treatment failure among adolescents living with HIV (ADLHIV) in a resource-limited setting (RLS), will provide relative time-estimates for treatment failure, and propose locally affordable measures to limit ART failure in ADLHIV; study objective 2 will provide the burden of drug resistance according to the national treatment guidelines, and would inform on the adequacy between detected mutations and the most potentially active second- or third-line drugs for ADLHIV in such RLS. This study objective is inform if the development of HIVDR are specific to the different local HIV-1 clades, for possible clinical considerations for a specific management of adolescents in the country; study objective 3 will inform on the potential compartmentalisation of HIVDR among adolescents, in order to design drug combinations that provide maximal efficacy in a long run; study objective 4 will identify both programmatic factors favouring HIVDR emergence among ADLHIV, among which events of ARV stock outs and the quality of dispensing practices. This same objective will also identify the level of adherence of ADLHIV to ART, among which non-observance, delay drug pick-up and lost to follow-up. Importantly, with the exception of settings where HIV-1 subtype C prevails (found mainly in Southern Africa), our study impacts could be generalizable to the population of ADLHIV in several RLS, giving potential endorsements by national and international health regulatory bodies such the World Health Organisation (WHO) and UNAIDS.

## Additional file

Additional file 1: Supplementary digital contents (SDC). SDC 1: Overall summary of major timeline and activities. (DOCX 29 kb)

## Data Availability

Not applicable.

## Abbreviations

ADLHIV: Adolescent living with HIV
AIDS: Acquired immunodeficiency syndrome
ART: Antiretroviral therapy
ARV: Antiretroviral
AZT: Zidovudine
BD: Becton Dickinson
CCR5: C-C chemokine receptor type 5
CD3: Cluster of differentiation 3
CD3: Cluster of differentiation 4
CD8: Cluster of differentiation 8
CIRCB: Chantal BIYA International Reference for research on HIV/AIDS prevention and management
CRF: Case reporting form
CRF: Circulating recombinant form
CXCR4: C-X-C motif chemokine receptor type 4
DNA: 2′-deoxy-ribose nucleic acid
DRM: Drug resistance mutation
EWI: Early warning indicators
FACS: Fluorescent activated cell sorting
GRT: genotypic resistance testing
HIV: Human immunodeficiency virus
HIVdb: HIV database
HIVDR: HIV drug resistance
IC: Interval confidence
ID: Identification
LPV/r: ritonavir-boosted lopinavir
LTFU: Lost to follow-up
M0: Month 0
M12: Month 12
M6: Month 6
NNRTI: Non-nucleoside reverse-transcriptase inhibitor
NRTI: Nucleoside reverse-transcriptase inhibitor
NVP: Nevirapine
PCR: Polymerase chain reaction
PMTCT: Prevention of mother-to-child transmission of HIV
PR-RT: Protease-reverse transcriptase
PVL: Plasma viral load
RLS: Resource-limited settings
RNA: Ribose nucleic acid
RT: Reverse transcriptase
RTI: Reverse transcriptase inhibitor
RTPCR: Reverse transcriptase polymerase chain reaction
SDC: Supplementary digital content
SSA: Sub-Saharan Africa
VF: Virological failure
WHO: World Health Organisation
