# Supplementary material for: Evaluation of treatment response, drug resistance and HIV-1 variability among adolescents on first- and second-line antiretroviral therapy: a study protocol for a prospective observational study in the centre region of Cameroon (EDCTP READY-study)
Source: BMC Pediatr. 2019 Jul 5;19:226. doi: 10.1186/s12887-019-1599-z (PMC6612130; doi:10.1186/s12887-019-1599-z)
Supplement: Supplementary file 1 — Supplementary digital contents (SDC). SDC 1: Overall summary of major timeline and activities. (DOCX 29 kb) [file 12887_2019_1599_MOESM1_ESM.docx]

**Supplementary digital contents (SDC)**

**SDC 1: Overall summary of major timeline and activities**

| **Activity per month-time** | **M1-M6** | **M7-M12** | **M13-M18** | **M18-M24** | **M24-M30** |
| --- | --- | --- | --- | --- | --- |
| **Mentorship working sessions** | **XX** | **XX** | **XX** | **XX** | **XX** |
| **Project preparadness** | **X** |  |  |  |  |
| **Workshop** |  |  |  |  | **X** |
| **Biostatistics/Epidemiology coursework** |  | **X** | **X** |  |  |
| **Research students** |  | **X** | **X** | **X** |  |
| **Enrolment of participants** |  | **X** |  |  |  |
| **HIV-RNA Viral load** |  | **X** | **X** | **X** |  |
| **CD4/CD8** |  | **X** | **X** | **X** |  |
| **Sanger sequencing*** |  | **X** | **X** | **X** |  |
| **Data entry** |  | **X** | **X** | **X** |  |
| **Data analysis** |  |  | **X** | **X** | **X** |
| **Scientific reporting** |  |  |  |  | **X** |
| **Conference and**  **EDCTP biennial meeting** |  |  |  |  | **X** |

**Sanger sequencing only if plasma viral load ≥1000 copies/ml. X means once; XX means twice.*

**Supplementary digital content 2:** study information sheets and informed consents

# Study Information Sheet

**(For the parent / legal guardian)**

**Title**: Evaluation of Treatment Response, Drug Resistance and HIV-1 Variability among Adolescents on First- and Second-Line Antiretroviral Therapy in Cameroon: The READY-Study

**Principal investigator**: Dr Joseph Fokam

**Invitation for your child to participate into a study on HIV management**

**Research objectives:** Among adolescents receiving antiretroviral treatment, we shall monitor the therapeutic response to first- and second-line regimens, drug resistance profiling and HIV-1 genetic variability during one-year follow-up.

**Target population**: Adolescents (10-19 years old) living with HIV receiving antiretroviral treatment

**Study period**: A period of 12 months constitutes the duration of participation.

**Study procedure**: After enrolment (month-0), follow-up will be done at mid-point (month-6) and at end-point (month-12), assessing CD4/CD8 and viral load response to antiretroviral treatment, including HIV-1 resistance testing in case of confirmed treatment failure. All laboratory results will be performed at the CIRCB (Chantal BIYA International Reference Centre for research on HIV/AIDS prevention and management) in Yaounde, and results will be provided free of charge to the participant’s physician for potential utility in his clinical management.

Residual samples will be conserved during five years maximum for eventual further investigations, and any relevant finding will be communicated to the study participant for his personal health benefit. After this storage period, samples will be discarded.

**Study participation**: You are free to allow your child’s participating into the study, and participation will remain voluntary throughout; you therefore have full right to refuse and withdraw your consent at anytime, without any coercion or without any implication on your child personal clinical management. A copy of your signed informed consent will be at your personal disposal.

**Requirements, benefits and risks of the study**: At each time point at the clinic, you may be ask to report on the child’s adherence and health; blood samples of 6ml will be drawn (venepuncture) by a qualified staff. Project staff will be responsible for transporting the sample for analysis at the CIRCB in Yaounde. Privacy and confidentiality will be ensured, through the use of unique identifiers and a protected computer and office, with access only to responsible project staffs. There is no payment provided for participation. As direct benefit, all laboratory tests will be covered, and results generated in the course of the study, including HIV-1 drug resistance testing, will be freely provided to each study participant to improve clinical management as per ARV drugs available locally for first-, second- and third-line regimens.

**For further details, you can contact the principal investigator**: Dr Joseph Fokam, Medical Virologist; Chantal BIYA International Reference Centre for research on HIV/AIDS prevention and management), Melen, Yaounde; telephone: 222235450; email: fokamjoseph@circb.cm.

**Address of the National Ethics Committee for Research on Human Health that issued ethical clearance for the study**: telephone: 243674339; email: [cnethique_minsante@yahoo.fr](mailto:cnethique_minsante@yahoo.fr)

**STUDY INFORMATION ABSTRACT**

**(For the adolescent)**

**Title**: Evaluation of Treatment Response, Drug Resistance and HIV-1 Variability among Adolescents on First- and Second-Line Antiretroviral Therapy in Cameroon: The READY-Study

**Principal investigator**: Dr Joseph Fokam

**Objectives**: As an adolescent receiving antiretroviral treatment, you are invited to participate in a study for your health response to treatment during one-year time.

**Participation**: You will come to the hospital clinic three (03) different times: At month-0, month-6 and month-12. Each time, you should report if you took daily your drugs “well” or “not”; blood samples equivalent to about three teaspoons will be collected and health results will be provided to improve your wellbeing. For five years maximum, we will keep your remaining sample and if there is any important result generated, this will be provided for your health benefit.

**Assent**: You are free to accept and you can also refuse at anytime. You will receive a copy of your acceptance letter that we advise to keep through your parent. You can also ask more information from your parent, or ask to contact the Doctor or the Committee that authorised the study.

# Notice d’information de l’étude

**(Pour le parent légal)**

**Titre**: Evaluation of de la Réponse Thérapeutique, la pharmaco-résistance du VIH-1, et Variabilité du VIH-1 chez les Adolescents sous Première et Deuxième Ligne de Traitement Antirétroviral au Cameroun: L’Etude «READY»

**Investigateur Principal**: Dr Joseph Fokam

**Invitation pour la participation de votre enfant à l’étude sur la prise en charge du VIH**

**Objectifs de l’étude:** Chez les adolescents recevant un traitement antirétroviral, nous évaluerons la réponse thérapeutique aux protocoles de première et de deuxième ligne, le profil de résistance aux antirétroviraux, et la variabilité génétique du VIH-1 durant un suivi d’un an.

**Population cible**: Adolescents (10-19 ans) vivant avec le VIH recevant un traitement antirétroviral.

**Période d’étude**: La durée est de 12 mois pour une participation complète.

**Déroulement de l’étude**: Après enrôlement (mois-0), un suivi sera effectué au mois-6 et au mois-12, évaluant le taux des CD4/CD8 et la charge virale, ainsi que le test de résistance génotypique du VIH-1 aux antirétroviraux en cas d’échec thérapeutique avéré. Tous les examens de laboratoire seront réalisés au CIRCB (Centre International de Référence Chantal BIYA pour la recherche sur la prévention et la prise en charge du VIH/SIDA) à Yaoundé, et les résultats seront fournis gratuitement au clinicien responsable du participant à but d’utilité clinique potentielle.

Le matériel biologique résiduel sera conservé pendant cinq au maximum, pour d’éventuelles investigations approfondies, et tout résultat probant sera communiqué au participant pour sa prise en charge bioclinique et son bien être personnel. Apres la période de conservation, tout échantillon biologique encore disponible sera détruit.

**Participation à l’étude**: Vous êtes entièrement libre d’autoriser la participation de votre enfant à l’étude, et cette participation sera toujours volontaire tout au long de l’étude; vous ainsi le plein droit de refuser ou de retirer votre consentement à tout moment, sans contrainte ni aucune implication sur la prise en charge de votre enfant. Une copie de votre consentement vous sera remise pour votre propre gouverne.

**Nécessités, bénéfices et risques liés à l’étude**: A chaque moment du suivi à la clinique, vous pourrez être appelé à fournir des informations sur l’adhérence de l’enfant et sa santé ; 6ml de sang veineux sera prélevé par un personnel qualifié. L’intimité et la confidentialité seront assurées à l’aide des codes d’identification et un bureau et ordinateur protégés d’accès aux seuls personnels responsables du projet. Un paiement pour participation n’est pas prévu. Comme bénéfice direct, les examens d’analyse seront pris en charge et les résultats, y compris le test de résistance du VIH-1, seront rendus gratuitement pour toute éventuelle optimisation de la prise en charge basée sur les ARV disponibles localement en première, deuxième et troisième ligne.

**Pour tout détail, contacter l’investigateur principal**: Dr Joseph Fokam, Virologue; Centre International de Référence Chantal BIYA pour la recherche sur la prévention et la prise en charge du VIH/SIDA) à Melen, Yaoundé; téléphone: 222235450; email: [fokamjoseph@circb.cm](mailto:fokamjoseph@circb.cm)

**Adresse du Comité National d’Ethique pour la Recherche en Santé Humaine, ayant délivre la clairance éthique pour l’étude**: téléphone: 243674339; email: [cnethique_minsante@yahoo.fr](mailto:cnethique_minsante@yahoo.fr)

**NOTICE D’INFORMATION SYNTHETIQUE**

**(Pour l’adolescent)**

**Titre**: Evaluation of de la Réponse Thérapeutique, la pharmaco-résistance du VIH-1, et Variabilité du VIH-1 chez les Adolescents sous Première et Deuxième Ligne de Traitement Antirétroviral au Cameroun: L’Etude «READY»

**Investigateur Principal**: Dr Joseph Fokam

**Objectifs**: Comme tout adolescent recevant un traitement antirétroviral, tu es invité(e) à participer à cette étude qui suivre ta réponse au traitement et ton état de sante pendant un an.

**Participation**: Tu viendras à l’hôpital trois (03) fois: Au mois-0, au mois-6 et au mois-12. Chaque fois, tu diras si tu as « bien » ou « mal » pris tes médicaments chaque jour; un prélèvement de sang équivalent à trois (3) cuillères à café sera collecté et les résultats de ton état de santé serviront a améliorer ton bien être. Durant cinq (5) années au maximum, nous garderons le reste de matériel, et si autre résultat important est obtenu, il sera mis à disposition pour ton bien être.

**Assentiment**: Tu es libre d’accepter ou de refuser à tout moment ta participation. Tu recevras une copie de la lettre de ton accord dont nous te conseillons de faire garder par ton parent. Tu peux aussi avoir des informations par ton parent, ou demander à contacter le Docteur ou le Comité qui a autorise l’étude.

# CONSENT FORM

**INFORMED CONSENT**

**(For the parent / legal guardian)**

I the undersigned, Mr/Mme/Mlle [Name(s) and surname(s)]:.………………………………………….., legal guardian of [Name(s) and surname(s)]:………………………………………………………………

Acknowledge to have been invited to provide a consent for the study entitled: “**Evaluation of Treatment Response, Drug Resistance and HIV-1 Variability among Adolescents on First- and Second-Line Antiretroviral Therapy in Cameroon: The READY-Study**”, of which the Principal Investigator is named Dr Joseph Fokam, Medical Virologist; Chantal BIYA International Reference Centre for research on HIV/AIDS prevention and management), Melen, Yaounde; telephone: 222235450; email: [fokamjoseph@circb.cm](mailto:josephfokam@gmail.com).

- I have read and well understood the study information sheet provided;
- Or the study information sheet have been read and explained to me;
- I have well understood the goals and objectives of the study;
- I have received all relevant answers to my questions regarding the study;
- The study requirements, risks and benefits have been presented and explained;
- I well understood that I am deliberately free to accept or refuse participation of my child;
- My consent does not discharge the study investigators from their responsibilities;
- I preserve all my rights, as well as those of my child, as per legal regulations.

Thus, I freely and voluntarily accept my child’s participation within the conditions mentioned in the study information sheet, namely:

- To provide medical information necessary for the child monitoring within the study;
- To watch and inform on the child’s adherence level and health status within study scope;
- To ensure the child’s attendance of planed clinic appointments for the study;
- To allow the collection of 6ml of blood for laboratory analyses;
- To ensure the appropriate use of laboratory results provided within the study;
- To authorise the storage of residual samples within the study for subsequent investigations, and to receive any relevant results thereof.

I therefore provide acceptance for my child’s participation to this study.

Place: ………………………………, on the……/……/……….

Study Site Principal Investigator Legal guardian of the participating adolescent

(Name and address) (Name and address)

Signature Signature

Of behalf of adolescent:…………………………..

**INFORMED ASSENT**

**(For the adolescent)**

I the undersigned, Mr/Mme/Mlle [Name(s) and surname(s)]:.…………………………………………..,

Acknowledge to have been invited to participate in the research study entitled: “**Evaluation of Treatment Response, Drug Resistance and HIV-1 Variability among Adolescents on First- and Second-Line Antiretroviral Therapy in Cameroon: The READY-Study**”, of which the Principal Investigator is named Dr Joseph Fokam, Medical Virologist; Chantal BIYA International Reference Centre for research on HIV/AIDS prevention and management), Melen, Yaounde; telephone: 222235450; email: [fokamjoseph@circb.cm](mailto:josephfokam@gmail.com).

- I have understood the study information sheet provided;
- Or the study information sheet have been read and explained to me;
- I have well understood the goals and objectives of the study;
- I have received all relevant answers to my questions regarding the study;
- The study requirements, risks and benefits have been presented and explained;
- I well understood that I am deliberately free to accept or refuse my participation;
- My assent does not discharge the study investigators from their responsibilities;
- I have all my rights according to the law.

Thus, I freely and voluntarily accept to participate within the conditions mentioned in the study information sheet, namely:

- To contribute in providing medical information necessary for monitoring within the study;
- To inform on my adherence level and health status within the scope of the study;
- To attend the planed clinic appointments within the scope of the study;
- To comply for the collection of 6ml of blood for laboratory analyses;
- To authorise the storage of residual samples within the study for subsequent investigations, and to receive any relevant results thereof.

I therefore provide acceptance for my child’s participation to this study.

Place: ………………………………, on the……/……/……….

Study Site Principal Investigator Participating

(Name and address) (Name and address)

Signature Signature

# FORMULAIRE DE CONSENTEMENT

**CONSENTEMENT PARENTAL ECLAIRE**

**(Pour le parent légal)**

Je soussigné, Mr/Mme/Mlle [Nom(s) et Prénom(s)] :.…………………………………………………….

Parent légal de [Nom(s) et Prénom(s)] :……………………………………………………………………

Reconnais avoir été invité à donner mon accord au travail de recherche intitulé «**Evaluation of de la Réponse Thérapeutique, la pharmaco-résistance du VIH-1, et Variabilité du VIH-1 chez les Adolescents sous Première et Deuxième Ligne de Traitement Antirétroviral au Cameroun: L’Etude ‘‘READY’’**», dont l’investigateur Principal s’appelle Dr Joseph Fokam, Virologue en service au Centre International de Référence Chantal BIYA pour la recherche sur la prévention et la prise en charge du VIH/SIDA) à Melen, Yaoundé; téléphone: 222235450; email: [fokamjoseph@circb.cm](mailto:josephfokam@gmail.com).

- J’ai lu et bien compris la notice d’information qui m’a été remise concernant l’étude ;
- Ou bien on m’a lu et expliqué la notice d’information relative à cette étude ;
- J’ai bien compris l’intérêt et les objectifs de cette étude ;
- J’ai reçu toutes les réponses aux questions que j’ai posées ;
- Les nécessités, risques et bénéfices liés à l’étude m’ont été présentés et expliqués ;
- J’ai bien compris que je suis libre d’accepter ou de refuser la participation de mon enfant ;
- Mon consentement ne décharge pas les investigateurs de la recherche de leurs responsabilités ;
- Je conserve tous mes droits, ainsi que ceux de mon enfant, garantis par la loi.

Ainsi, j’accepte librement la participation de mon enfant dans les conditions précisées dans la notice d’information de l’étude, notamment :

- De fournir les informations médicales nécessaires pour son suivi durant l’étude ;
- De veiller et d’informer du niveau d’adhérence et de l’état de santé de l’enfant durant la période de l’étude ;
- De veiller aux respects des rendez-vous programmés;
- D’autoriser le prélèvement de 6ml de sang pour les analyses de laboratoire ;
- De veiller au bon usage des résultats fournis dans le cadre de l’étude ;
- D’accorder que le reste des échantillons prélevés dans le cadre de cette étude soit conservé pour des études ultérieures, et que tout utile me soit communiqué.

Je donne donc mon accord pour ma participation à cette étude.

Fait à………………………………, le……/……/……….

Investigateur Principal du site d’étude Parent légal

(Nom et adresse) (Nom et adresse)

Signature Signature

Pour l’adolescent(e):…………………………………

**ASSENTIMENT ECLAIRE**

**(Pour l’adolescent)**

Je soussigné, [Nom(s) et Prénom(s)] :.…………………………………………………….

Reconnais avoir été invité à participer au travail de recherche intitulé «**Evaluation of de la Réponse Thérapeutique, la pharmaco-résistance du VIH-1, et Variabilité du VIH-1 chez les Adolescents sous Première et Deuxième Ligne de Traitement Antirétroviral au Cameroun: L’Etude ‘‘READY’’**», dont l’investigateur Principal s’appelle Dr Joseph Fokam, Virologue en service au Centre International de Référence Chantal BIYA pour la recherche sur la prévention et la prise en charge du VIH/SIDA) à Melen, Yaoundé; téléphone: 222235450; email: [fokamjoseph@circb.cm](mailto:josephfokam@gmail.com).

- J’ai lu et bien compris la notice d’information qui m’a été remise concernant l’étude ;
- Ou bien on m’a lu et expliqué la notice d’information relative à cette étude ;
- J’ai bien compris l’intérêt et les objectifs de cette étude ;
- J’ai reçu toutes les réponses aux questions que j’ai posées ;
- Les nécessités, risques et bénéfices liés à l’étude m’ont été présentés et expliqués ;
- J’ai bien compris que je suis libre d’accepter ou de refuser d’y participer;
- Mon assentiment ne décharge pas les investigateurs de la recherche de leurs responsabilités ;
- Je conserve tous mes droits garantis par la loi.

J’accepte librement de participer dans les conditions précisées dans la notice d’information de l’étude, notamment :

- De contribuer à fournir les informations médicales nécessaires pour son suivi durant l’étude ;
- D’informer de mon adhérence et de mon état de santé durant la période de l’étude ;
- De veiller aux respects de mes rendez-vous de suivi programmés;
- D’accepter le prélèvement de 6ml de sang pour les analyses de laboratoire ;
- D’accorder que le reste des échantillons prélevés dans le cadre de cette étude soit conservé pour des études ultérieures, et que tout utile me soit communiqué.

Je donne donc mon accord pour ma participation à cette étude.

Fait à………………………………, le……/……/……….

Investigateur Principal du site d’étude Participant

(Nom et adresse) (Nom et adresse)

Signature Signature
